# Why primary care practices should become digital health information hubs for their patients

**DOI:** 10.1186/s12875-014-0190-9

**Published:** 2014-11-25

**Authors:** Aaron Baird, Samantha Nowak

**Affiliations:** Institute of Health Administration and Department of Computer Information Systems, J. Mack Robinson College of Business, Georgia State University, PO Box 3988, Atlanta, Georgia 30302-3988; Dunwoody Pediatrics, 1428 Dunwoody Village Pkwy, Dunwoody, GA 30338 USA

**Keywords:** Primary care, Patient-facing technologies, Health information hubs

## Abstract

**Background:**

Two interesting health care trends are currently occurring: 1) patient-facing technologies, such as personal health records, patient portals, and mobile health apps, are being adopted at rapid rates, and 2) primary care, which includes family practice, is being promoted as essential to reducing health care costs and improving health care outcomes. While these trends are notable and commendable, both remain subject to significant fragmentation and incentive misalignments, which has resulted in significant data coordination and value generation challenges. In particular, patient-facing technologies designed to increase care coordination, often fall prey to the very digital fragmentation issues they are supposed to overcome. Additionally, primary care providers are treating patients that may have considerable health information histories, but generating a single view of such multi-source data is nearly impossible.

**Discussion:**

We contribute to this debate by proposing that primary care practices become digital health information hubs for their patients. Such hubs would offer health data coordination in a medically professional setting with the benefits of expert, trustworthy advice coupled with active patient engagement. We acknowledge challenges including: costs, information quality and provenance, willingness-to-share information and records, willingness-to-use (by both providers and patients), primary care scope creep, and determinations of technical and process effectiveness. Even with such potential challenges, we strongly believe that more debate is needed on this topic prior to full implementation of various health information technology incentives and reform programs currently being designed and enacted throughout the world. Ultimately, if we do not provide a meaningful way for the full spectrum of health information to be used by both providers and patients, especially early in the health care continuum, effectively improving health outcomes may remain elusive.

**Summary:**

We view the primary care practice as a central component of digital information coordination, especially when considering the current challenges of digital health information fragmentation. Given these fragmentation issues and the emphasis on primary care as central to improving health and lower overall health care costs, we suggest that primary care practices should embrace their evolving role and should seek to become digital health information hubs for their patients.

## Background

We reflect on why patient usage of patient-facing technologies is often limited and why health outcomes are difficult to impact with patient-facing technologies [1-5]. We believe this to be an especially important debate given: 1) the increasing importance of primary care in improving health and lowering health care costs, 2) the increasing information intensity in health care, 3) and the ongoing efforts throughout the world to leverage digital health information to improve care coordination and health outcomes (e.g., “meaningful use” EHR incentives in the U.S., the Digital Agenda for eHealth within the E.U., care reform policies taking place in the U.K., etc.^a^) [6-8]. We propose a specific solution that we hope will contribute to meaningful debate. We propose that primary care providers should become *digital health information hubs for their patients* and should be able to use combinations of practice-generated information, patient-generated information (when given permission), and any other medically relevant information, often from imported from other devices or digital health services, to provide more informed and personalized health recommendations.

Current use of patient-facing technologies is often limited because of the fragmentation of this market, the difficulty in sharing digital information with providers, and the lack of comprehensive value generated when attempting to use multiple patient-facing technologies [5,9]. In particular, uptake of patient-facing technologies is often dependent on specific patient needs (e.g., vulnerability to chronic disease) [10], is typically primarily focused on patient convenience and self-service capabilities (e.g., viewing lab results and scheduling appointments) [11] as well as supply-side operational efficiencies [1], supply-side adoption is often contingent on regional externalities [12], and policies do not always keeps pace with this dynamic market [13]. What is needed are digital services that can securely store and analyze the majority of our health information, aggregated from multiple sources (tagged appropriately with provenance meta-data, see [9] for more details), with the ability to share this information with health care providers toward the goal of generating informed insights and, ultimately, improving health. While the value of using such aggregated data in care coordination processes has been touted in the research literature [14-16] and in government-sponsored reports speaking to the value of aggregated, multi-sourced information in care delivery processes [17,18], digital information hubs that fully intermediate the patient-provider relationship in the primary care setting are not yet widely available.

Many efforts have been made toward the overall goal of using data to facilitate care coordination, but, unfortunately, these attempts are often significantly fragmented, especially from the point-of-view of the patient [5]. We acknowledge that many initiatives are underway to improve health information exchange and care coordination [9,17,19,20], but suggest that many such initiatives are often provider-centric rather than patient-centric [5]. Additionally, it has also been recommended to place more data under patient control as health information exchange policies are formed [9], which we agree with, but we also question whether or not patients, especially those who are on the more extreme ends of the continuum—ranging from those who only use health care in emergencies to those under regular medical care and supervision—will be willing and able to take on the needed responsibility to actively manage their own data. Therefore, we suggest that our proposal balances provider-centric and patient-centric digital health data management incentives and challenges.

## Discussion

Consider the example of patient portals, a key technology being incentivized by meaningful use incentive policies in the U.S. [21,22], by the Digital Agenda policies in the E.U. [7], and by health care reform efforts in the U.K. [17]. A patient portal is defined by HealthIT.gov, the communication web site for the U.S. Office of the National Coordinator (ONC) for Health Information Technology, as “a secure online website that gives patients convenient 24-hour access to personal health information from anywhere with an Internet connection [23]”. A tethered patient portal is tied directly to a medical practice and provides convenient access to information and digital services at that practice. HealthIT.gov goes on to say, “With patient portal implementation, your organization can enhance patient-provider communication, empower patients, support care between visits, and, most importantly***,*** improve patient outcomes [23]”. Yet, several studies have shown that while patient-provider communication is often improved and more efficient after patient portals are introduced, impacts on health outcomes are difficult to demonstrate, except in very specific contexts or chronic disease care settings [1,2]. A recent paper did show that four mechanisms of patient portals are especially likely to impact outcomes associated with patient portal usage—insight into personal health information, activation of information, interpersonal continuity of care, and service convenience—but also stated that patient portals seem to be the most effective in integrated health service networks [24]. We interpret the success of patient portals in U.S. integrated health service networks as partly due to the fact that information is seamlessly shared between in-network providers (and also that more complex features are likely less costly to implement when economies-of-scale are involved) [1]. Interestingly, even the highly touted Blue Button initiative^b^ often associated with the U.S. Veteran’s Health Administration (VHA) myHealtheVet patient portal, allows for export of VHA data for sharing with other providers and other digital health services, but not for import of health information from outside sources into myHealtheVet. It is incumbent on the patient to find a third-party service to import the data into [25,26]. Generally speaking, therefore, patient portals seem to be often designed to either store and analyze basic health information (e.g., Microsoft HealthVault) or to help a provider meet requirements (e.g., U.S. meaningful use requirements) [2,27].

If we extend the example of patient portals to recent innovations in the area of patient-generated health data, limitations and information fragmentation remain. While there have been some examples of successful integration of EHRs and patient-generated data [28], patient telehealth data [29], and diabetes self-management data [30], the majority of patient-facing technologies are not necessarily designed to provide comprehensive, coordinated value to patients via patient-provider linkages. In particular, patient-facing technologies are more often designed to meet the needs of the suppliers (providers) and provide basic conveniences to consumers, but not necessarily to improve overall health (or, at least, it is incumbent on the health care consumer to coordinate various and disparate sources of health information and digital health features). If the design of the technology is primarily centered on meeting policy guidelines and patient convenience, it is not surprising that making a direct connection between patient-facing technologies and health outcomes is often challenging. We argue that the *potential* benefits of such technologies are indisputable, but *realizing* such benefits often requires patients to be dedicated to a single integrated delivery network of health care providers and services, or to have the willingness to spend a significant amount of personal time and effort to aggregate and manage fragmented information; both of which are often unrealistic requirements.

To resolve this issue, we propose a solution that we admit would be difficult to implement in practice, but one that would provide real value to patients of all sorts, ranging from those who are mostly healthy and visit providers sporadically (or mostly for annual visits) to those with complex, chronic conditions who visits multiple providers regularly. We specifically propose that *primary care providers become digital health information hubs for their patients*. In particular, primary care digital health information hubs would provide patients with direct access to health information from the primary care practice they visit, would allow patients and providers to import health information from other sources (e.g., interoperable provider-generated medical records and patient-generated health data as well as any other relevant data from other digital health services), and would permit patients to grant or deny provider access to information imported from external sources. In such a *primary care digital health information hub* model, health information fragmentation could be significantly reduced while also giving patients the opportunity to leverage aggregated, digital information when seeking medical advice, treatment, and insights. Improving health outcomes and enhancing patient control of personal health information would be central to the value-proposition in this proposal. In the future, if additional information is eventually included, such as medication adherence or treatment compliance, as could be enabled by other technologies, primary care providers could further enhance diagnostic, treatment, and referral recommendations based on truly coordinated and digitized information. Such a model would be more centralized for individual patients, especially as primary care is becoming elevated in importance in the health care continuum, and would also retain the benefits of decentralization from a provider’s point-of-view, as aggregated information would be often be more local rather than global in scope. Additionally, the incentives for both providers and patients could emphasize health and health outcomes, key goals of many current health reform efforts e.g. [17,31], over profit and lock-in motives often observed in the health information industry [5,9].

Challenges to this proposal would include: *costs* (especially given that money flows to primary care are often limited), *information quality and provenance* (maintaining information reliability, validity, and the “chain of custody”), *willingness-to-share* (other providers of health care, other providers of digital health services, and mHealth apps and medical device developers would all need to be convinced to share information with primary care providers), *willingness-to-use* (both providers and patients would have to make the time to manage information, keep it up to date, and use the information when making decisions), *primary care scope creep* (as primary care practice scope would increase to include information technology service provisioning and management as well as evaluation of aggregated health information), and determinations of *effectiveness* (effective use would likely require trial periods and an ongoing adaptive approach to process and technology design).^c^ In addition, developing and implementing such hubs would take time and effort that primary care physicians are in limited supply of. Finally, we would hope that such hubs would not be used primarily to “lock-in” patients, as is often observed in technology implementation and usage contexts [5,9]. Rather, we would hope that primary care providers would emphasize health outcomes and would embrace and encourage information sharing and interoperability, even when patients want to switch primary care providers (or have to switch, as when they move to another location).

## Summary

We view the primary care practice as a central component of digital health information sharing and coordination. We also recognize the increasing importance of primary care in improving health and lowering costs. To address digital fragmentation, incentive, and health outcome issues, we specifically propose that primary care practices become digital health information hubs for their patients. Such an approach would offer significant benefits to primary care providers often at an informational disadvantage due to currently less than optimal amounts of patient information during time-constrained visits. Such an approach would also significantly enhance the value proposition of patient-facing technologies for patients themselves, as health information would reside with an entity capable of offering individualized and evidence-based advice and treatment recommendations. We acknowledge that success likely rests on government support of primary care digital health hub initiatives, implementations that involve prototypes at first and phased approaches as time progresses, and significant interactions and adaptations coordinated with patients as value propositions evolve. While many challenges are likely and potential options are plentiful, just as there are many challenges and options with the adoption and diffusion of electronic health records, we propose that applying the triple aim (low cost, better care, better health) [31] to the context of patient-facing technologies requires that primary care providers embrace their role as hubs of health information in a health care system that cannot afford digital fragmentation. The ultimate result could be a marriage of an essential health service (primary care) and a family of technologies with a lot of potential (patient-facing technologies) that, together, could yield actual patient value.

## Endnotes

^a^For a comprehensive summary of health information technology progress and initiatives as well as and care coordination variation across developed countries, please see [19].

^b^The Blue Button initiative in the U.S. provides standardized health information file formats and a recognizable icon (a blue download button) that can be utilized by patients when seeking to download their medical records and information and, often, transfer that information to another provider or third-party.

^c^Additional technical, social, legal, and policy challenges are outlined in [18].

## Abbreviations

app: mHealth application
mHealth: Mobile health
PCMHs: Patient Centered Medical Homes
VHA: Veteran’s health administration

## References

[1] Emont S (2011). Measuring the Impact of Patient Portals: What the Literature Tells Us.

[2] Ammenwerth E, Schnell-Inderst P, Hoerbst A (2012). The impact of electronic patient portals on patient care: a systematic review of controlled trials. J Med Internet Res.

[3] Kellermann AL, Jones SS (2013). What it will take to achieve the as-yet-unfulfilled promises of health information technology. Health Aff.

[4] Jones SS, Rudin RS, Perry T, Shekelle PG (2014). Health information technology: an updated systematic review with a focus on meaningful use. Ann Intern Med.

[5] Oram A (2014). The Information Technology Fix for Health.

[6] Phillips RL, Brundgardt S, Lesko SE, Kittle N, Marker JE, Tuggy ML, LeFevre ML, Borkan JM, DeGruy FV, Loomis GA (2014). The future role of the family physician in the United States: a rigorous exercise in definition. Ann Fam Med.

[7] Commission to the European Parliment, The Council, the European Economic and Social Committee and the Committee of the Regions (2012). eHealth Action Plan 2012-2020 - Innovative Healthcare for the 21st Century.

[8] Blaya JA, Fraser HS, Holt B (2010). E-health technologies show promise in developing countries. Health Aff.

[9] McMorrow D (2014). A Robust Health Data Infrastructure.

[10] Rai A, Chen L, Pye J, Baird A (2013). Understanding determinants of consumer mobile health usage intentions, assimilation, and channel preferences. J Med Internet Res.

[11] Baird A, Raghu TS, North F, Edwards F (2014). When traditionally inseparable services are separated by technology: the case of patient portal features offered by primary care providers. Health Syst.

[12] Baird A, Furukawa M, Raghu TS (2012). Understanding contingencies associated with the early adoption of customer-facing web portals. J Manag Inform Syst.

[13] Baird A, Raghu TS, Tulledge-Scheitel SM (2012). The role of policy in the prevention of Personal Health Record (PHR) market failure. J Inform Technol Polit.

[14] Murdoch TB, Detsky AS (2013). The inevitable application of big data to health care. JAMA.

[15] Howie L, Hirsch B, Locklear T, Abernethy AP (2014). Assessing the value of patient-generated data to comparative effectiveness research. Health Aff.

[16] Bauer AM, Thielke SM, Katon W, Unützer J, Areán P (2014). Aligning health information technologies with effective service delivery models to improve chronic disease care. Prev Med.

[17] Hunt J, Stevens S (2014). Transforming Primary Care: Safe, Proactive, Personalised Care for Those Who Need It Most. Book Transforming Primary Care: Safe, Proactive, Personalised Care for Those Who Need it Most (Editor ed.^eds.).

[18] Shapiro M, Johnston D, Wald J, Mon D (2012). Patient-Generated Health Data. White paper: Prepared for Office of Policy and Planning.

[19] Thomson S, Osborn R, Squires D, Jun M (2013). International profiles of health care systems 2013, report number: 1717. The Commonwealth Fund.

[20] Furukawa MF, Patel V, Charles D, Swain M, Mostashari F (2013). Hospital electronic health information exchange grew substantially in 2008–12. Health Aff.

[21] Ahern DK, Woods SS, Lightowler MC, Finley SW, Houston TK (2011). Promise of and potential for patient-facing technologies to enable meaningful use. Am J Prev Med.

[22] Ricciardi L, Mostashari F, Murphy J, Daniel JG, Siminerio EP (2013). A national action plan to support consumer engagement via E-Health. Health Aff.

[23] **What is a patient portal?** [http://www.healthit.gov/providers-professionals/faqs/what-patient-portal]

[24] Otte-Trojel T, de Bont A, Rundall TG, van de Klundert J (2014). How outcomes are achieved through patient portals: a realist review. J Am Med Inform Assoc.

[25] Turvey C, Klein D, Fix G, Hogan TP, Woods S, Simon SR, Charlton M, Vaughan-Sarrazin M, Zulman DM, Dindo L (2014). Blue Button use by patients to access and share health record information using the Department of Veterans Affairs’ online patient portal. J Am Med Inform Assoc.

[26] Haggstrom DA, Saleem JJ, Russ AL, Jones J, Russell SA, Chumbler NR (2011). Lessons learned from usability testing of the VA’s personal health record. J Am Med Inform Assoc.

[27] Steinbrook R (2008). Personally controlled online health data-the next big thing in medical care?. N Engl J Med.

[28] Marquard JL, Garber L, Saver B, Amster B, Kelleher M, Preusse P (2013). Overcoming challenges integrating patient-generated data into the clinical EHR: Lessons from the CONtrolling Disease Using Inexpensive IT–Hypertension in Diabetes (CONDUIT-HID) Project. Int J Med Inform.

[29] Davidson E, Simpson CR, Demiris G, Sheikh A, McKinstry B (2013). Integrating telehealth care-generated data with the family practice electronic medical record: qualitative exploration of the views of primary care staff. Interact J Med Res.

[30] Peeples MM, Iyer AK, Cohen JL (2013). Integration of a mobile-integrated therapy with electronic health records: lessons learned. J Diabetes Sci Technol.

[31] Berwick DM, Nolan TW, Whittington J (2008). The triple aim: care, health, and cost. Health Aff.

